# R package for animal behavior classification from accelerometer data—rabc

**DOI:** 10.1002/ece3.7937

**Published:** 2021-08-20

**Authors:** Hui Yu, Marcel Klaassen

**Affiliations:** ^1^ Centre for Integrative Ecology School of Life and Environmental Sciences Deakin University Geelong Vic Australia; ^2^ Druid Technology Co., Ltd. Chengdu China

**Keywords:** accelerometer, animal behavior classification, data visualization, interactive process, XGBoost

## Abstract

Increasingly, animal behavior studies are enhanced through the use of accelerometry. To allow translation of raw accelerometer data to animal behaviors requires the development of classifiers. Here, we present the “rabc” (**r** for **a**nimal **b**ehavior **c**lassification) package to assist researchers with the interactive development of such animal behavior classifiers in a supervised classification approach. The package uses datasets consisting of accelerometer data with their corresponding animal behaviors (e.g., for triaxial accelerometer data along the x, y and z axes arranged as “x, y, z, x, y, z,…, behavior”). Using an example dataset collected on white stork (*Ciconia ciconia*), we illustrate the workflow of this package, including accelerometer data visualization, feature calculation, feature selection, feature visualization, extreme gradient boost model training, validation, and, finally, a demonstration of the behavior classification results.

## Graphical abstract

Other than the serial functions to turn raw accelerometer data into behaviors, this package also provides interactive visualization tools to assist in handling and interpreting the accelerometer input data, deciding on appropriate behavior categories for classification and understanding the classification results. In brief, this package promotes the integration of the user's expert knowledge on their own research system in developing advanced behavior classification models.

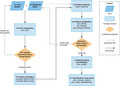

## 1 INTRODUCTION

Our understandings of animal movement patterns and behaviors continue to rapidly advance with the use of ever smarter and smaller tracking technologies (Ropert‐Coudert & Wilson, 2005; Williams et al., 2019). Increasingly, the tracking of animals is also combined with accelerometer (ACC) data collection to study the free‐roaming behaviors of animals across a wide range of taxa (Brown et al., 2013; Shepard et al., 2008). Compared with direct human observation, using ACC to study animal behaviors has the obvious advantage that it reduces the influence of human presence and also allows the recording of behaviors that would otherwise be hard to observe, away from the human eye (Brown et al., 2013). However, these obvious merits of ACC technology can only be achieved when a reliable behavior classification model is available that can convert ACC data into meaningful behavior types.

Many studies have already conducted behavior classification from ACC data (e.g., Nathan et al., 2012). In most cases, ACC data with corresponding behavioral field observations are used to train behavior classification models (e.g., Kölzsch et al., 2016; Kröschel et al., 2017). However, in some instances the developed classifiers that translate ACC data into behavior types yield only low classification accuracy (Fehlmann et al., 2017). As a general remedy, using fewer behavior classes and aggregating behaviors usually yields better classification performance (Ladds et al., 2017). Such grouping of behaviors is typically based solely on biological or ecological considerations without the use of computational pattern recognition. Nevertheless, some behaviors whose discrimination may have little biological value might have very similar ACC recording patterns and grouping of these behaviors based on the observed patterns might potentially yield better classification models. It is this often iterative process of grouping and splitting behaviors within the behavior set that the here presented rabc (**r** for **a**nimal **b**ehavior **c**lassification) package also endeavors to assist with. In this way, the rabc package allows the user to derive optimal and validated behavior classifiers suited to their specific research system and questions.

To help biologists translate ACC data into behaviors, this package uses XGBoost, which is currently one of the most promising supervised machine learning methods for this specific purpose (Yu et al., 2021). Unlike the web‐based tool “AcceleRater” (Resheff et al., 2014), our rabc package does not focus on providing a “one‐stop service” turning ACC data into behaviors. Rather, this package focuses on (a) providing interactive visualization tools to its user to assist in handling and interpreting the ACC input data, (b) deciding on appropriate behavior categories for classification as highlighted in the previous paragraph, and (c) reducing ACC data volume efficiently and effectively (through the calculation and selection of a range of features) without compromising behavior classification performance. In brief, this package endeavors to open the lid of the machine learning "black‐box", allowing the integration of the user's expert knowledge on their own research system in developing advanced behavior classification models.

## 2 rabc WORKFLOW

**FIGURE 1:**
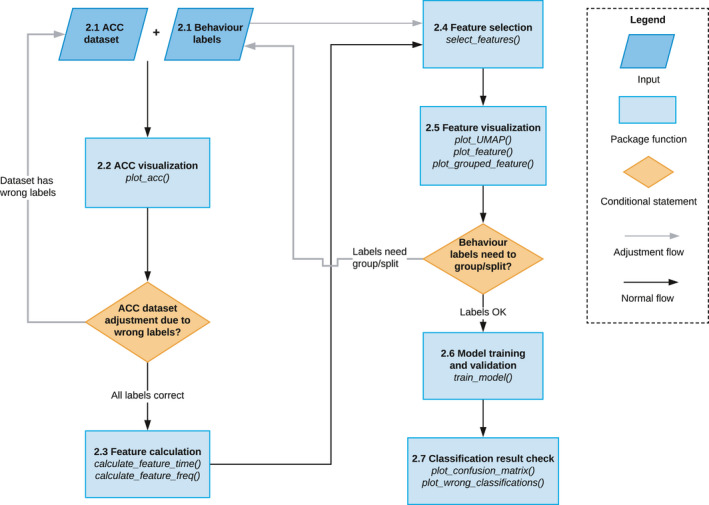
The general workflow of the rabc package to develop classifiers for adequately transforming ACC data into behaviors. The various elements in the diagram are numbered according to the paragraphs where these are being described in detail

The general workflow of the rabc package to transform ACC data using supervised machine learning methods into behaviors is outlined in Figure 1, Table 1. The data flow is composed of the following elements (where the numbering refers to the sections where these are being described in detail): 2.1 ACC dataset preparation with behavior labels; 2.2 ACC visualization; 2.3 Feature calculation; 2.4 Feature selection; 2.5 Feature visualization; 2.6 Model training and validation; and 2.7 Classification result check. Each section includes details on the use of the rabc package, including example code and results. The rabc package can be installed in Rstudio by “devtools::install_github(“YuHuiDeakin/rabc”, build_vignette=TRUE)”.

**TABLE 1:** Summary of rabc functions. In the “Wrapper” field, functions from other R packages that are being used in the rabc package are being listed

Functions	Arguments	Description	Wrapper
order_acc()	df_raw = NULL	Arrange the rows according to behavior labels	dplyr::arrange
plot_acc()	df_raw = NULL, axis_num = 3	Use dygraph to plot all accelerometer data grouped by behavior types	dygraphs::dygraph
calculate_feature_time()	df_raw = NULL, winlen_dba, axis_num = 3	Calculate accelerometer data into time domain mathematical features	
calculate_feature_freq()	df_raw = NULL, samp_freq, axis_num = 3	Calculate accelerometer data into frequency domain features	
feature_selection()	df_feature = NULL, vec_label = NULL, filter = FALSE, cutoff = 0.9, wrapper = "XGBoost", no_features = 5	Select a subset of relevant features for use in behavior classification	caret::train
plot_selection_accuracy()	results = NULL	Plot accuracies of selected features during feature selection procedures	ggplot2::ggplot
plot_feature()	df_feature = NULL, vec_label = NULL	Use dygraph to plot feature(s) in sequence grouped by behavior types	dygraphs::dygraph
plot_grouped_feature()	df_feature = NULL, vec_label = NULL, geom = "boxplot"	Plot feature distributions grouped by behavior types	ggplot2::ggplot
plot_UMAP()	df_time = NULL, df_freq = NULL, label_vec = NULL	Plot two‐dimensional UMAP that embedding high dimensional features	umap::umap; ggplot2::ggplot
train_model()	df_feature = NULL, vec_label = NULL, hyper_choice = "defaults", train_ratio = 0.75	XGBoost model training and validation	caret::train
plot_confusion_matrix()	df_feature = NULL, vec_label = NULL	Plot classification‐result confusion table	caret::train
plot_wrong_classifications()	df_raw = NULL, axis_num = 3, df_result = NULL	Use dygraph to plot wrong classification bouts on all acceleration data	dygraphs::dygraph

### 2.1 ACC dataset preparation and behavior labels

Segments of continuous ACC data will need to be translated into meaningful behaviors. For ACC data segmentation, there are two choices: even‐length segmentation and variable‐length segmentation (Bom et al., 2014). Variable‐length segmentation requires an algorithm to detect behavior change points and may thus be prone to error. Even‐length segmentation does not require these additional calculations and is therefore much easier to implement. However, even‐length ACC segments will inevitably contain behavior change points (and thus multiple behaviors) affecting down the line processing and behavior classification. An ACC segment should be sufficiently long to contain enough data to be representative of a behavior (and, thus, interpretable as a specific behavior type), whereas its length should be limited to avoid inclusion of multiple behaviors as much as possible. Regarding the inevitable segments where behavior transitions take place, we recommend retaining these segments in the model training. Although these data might decrease the accuracy of the classification model, they will make the model more robust and avoid overestimating model performance.

The rabc package only supports even‐length segmentation data with corresponding behavioral data, that is, the key behavior scored for the duration of the segment. These behavioral data are essential for supervised machine learning methods. ACC data collection with associated behavioral observations can be made both in the wild (e.g., Kröschel et al., 2017) and in captivity (e.g., Kölzsch et al., 2016). Obviously great care should be taken that observations (video recording) are accurately synchronized with ACC data collection (e.g., Kröschel et al., 2017). Although not provided by the rabc package, to reduce signal noise raw ACC data can potentially be preprocessed (Brown et al., 2013) before entering the data into the rabc package. Such “filtered” data would however require that any behavioral classification model generated by the rabc package is used to predict behaviors on filtered ACC data exclusively.

The input data should be a data.frame or tibble containing data including the behavior associated with the ACC data. For triaxial ACC data, each row of equal length should be arranged as "x, y, z, x, y, z, …, behavior", where “behavior” is the (primary) behavior observed during that segment. For dual‐axial ACC data, it should be arranged as "x, y, x, y, …,behavior" and for single‐axial ACC data as "x, x, …, behavior". A range of ACC data formats exist that are different to the format required by the rabc package. For instance, ACC data from triaxial trackers developed by e‐obs GmbH (Munich, Germany) are arranged as “x y z x y z … ”. At the end of this section, we provide an example for reading data recorded by e‐obs trackers (Pokrovsky et al., 2021) and transforming these into a format suitable for the rabc package. Data provided by Ornitela (Vilnius, Lithuania) and Druid Technology (Chengdu, China) ACC trackers are arranged in a four column table format, where each row contains “timestamp, x, y, z”. Thus, 10 rows of data make one second of ACC recordings when the sampling frequency is 10 Hz. In the vignette of the rabc package, which can be accessed by using the function “browseVignettes(‘rabc’)”, we provide an example on converting this specific format to the format required by the rabc package.

The here used triaxial ACC demo dataset from white stork (*Ciconia ciconia*) (data accessible from the AcceleRater website: http://accapp.move‐ecol‐minerva.huji.ac.il/, see Resheff et al., 2014) was measured at 10.54 Hz. Forty triaxial measurements, totaling 3.8 s, were used to form a behavior segment. The dataset includes 1,746 segments each forming a row in the dataset. Each row contains 121 columns. The first 120 columns are ACC measurements from three orthogonal axes, arranged as x, y, z, x, y, z, …,x, y, z. The final column is of type character containing the corresponding behavior. The dataset contains 5 different behaviors including "A_FLIGHT" ‐ active flight (77 cases), "P_FLIGHT" ‐ passive flight (96), "WALK" ‐ walking (437), "STND" ‐ standing (863), "SITTING" ‐ sitting (273).

In the following the relevant R code reading and converting ACC data:

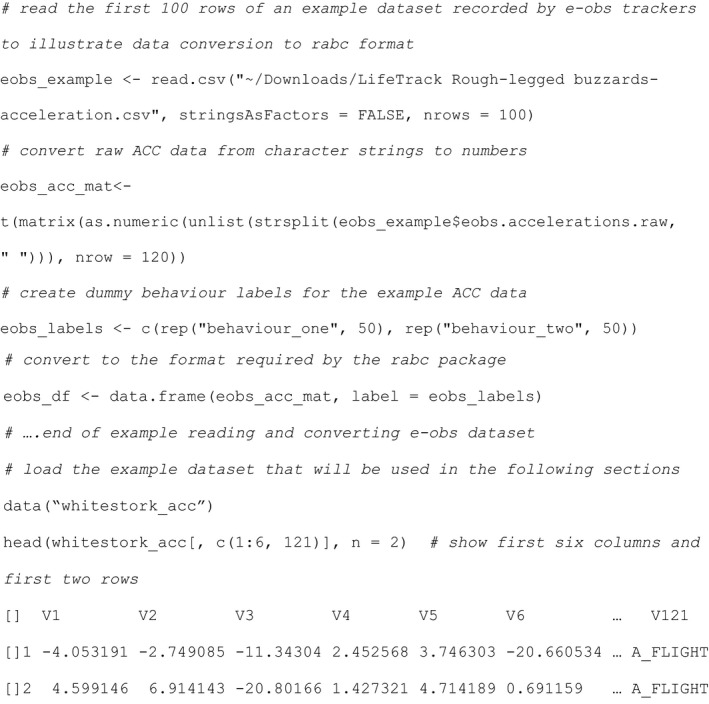

### 2.2 ACC visualization

The rabc package offers two types of graphs, that is, dynamic graphs and static graphs. Dynamic graphs produced by the “dygraphs” package (Vanderkam et al., 2018) allow users to zoom in and out and scroll through the depicted ACC data to facilitate data examination. Static graphs produced by the “ggplot2” package (Wickham, 2016) help users to examine feature distributions and to check behavior classification results.

Prior to visualizing the ACC data, the dataset needs to be sorted by behavior using the order_acc function. The purpose of this function is to ease comparison of ACC patterns among segments sharing the same behavior labels. For ACC data visualization, the rabc package uses the function dygraph from the “dygraphs” package to plot all ACC segments grouped by behavior. This dynamic mode of presentation provides the user with a visual impression of how the ACC signal generally relates to the different behaviors and can also be used for data quality control (i.e., identifying potentially incorrect segments where ACC and behavioral data do not conform to the general pattern otherwise observed due to, for instance, incorrect behavioral observation). The x‐axis of this dygraph indicates the row sequence number (i.e., the segment number) of the sorted data.

**FIGURE 2:**
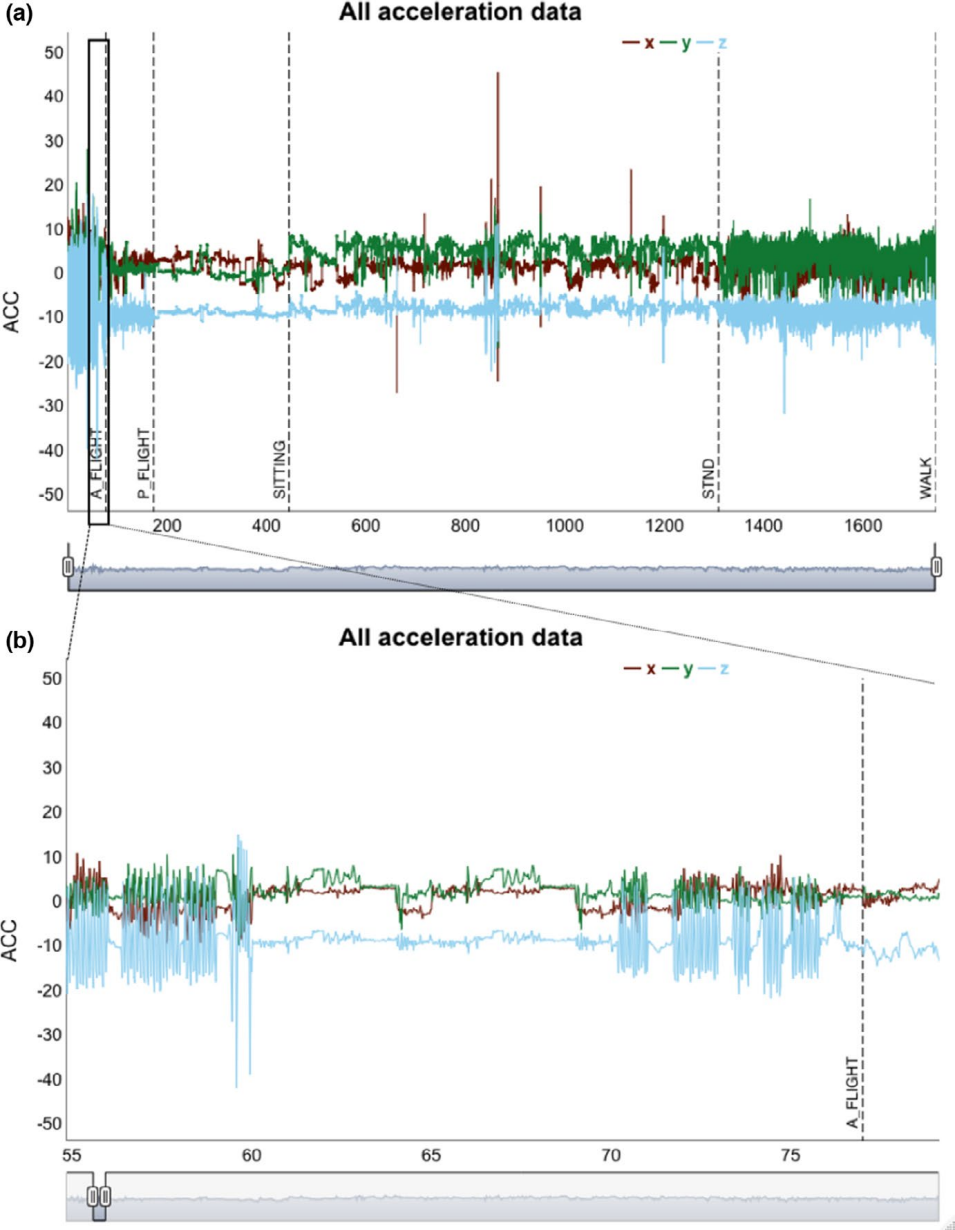
ACC data visualization using a dynamic graph. Panel a shows the complete white stork ACC dataset, sorted by behavior type. The x‐axis shows the segment numbers of the dataset ordered by behavior. Vertical dashed lines separate different behavior types. Panel b demonstrates how one can zoom in on specific segment ranges, here from segment 55 to 80

Plotting the complete white stork ACC dataset using function plot_acc (Figure 2a) and next zooming in on the area around segments 55–80 (Figure 2b), it can be seen that the ACC data between segments 60 and 70 is very different from neighboring segments. Albeit all being labeled as “A_FLIGHT”, the ACC data in this range resemble more static behaviors, warranting their scrutiny and, potentially, their relabeling or removal from the dataset.

In the following, the relevant R code plotting ACC data:

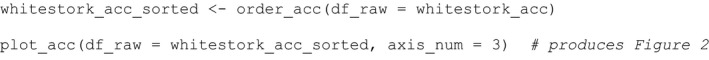

### 2.3 Feature calculation

The next step is to calculate features from the ACC data. A feature is a specific mathematical description (such as the mean and the standard deviation) of the ACC signal within a segment, which will form the input to the machine learning models (Brown et al., 2013). Using functions calculate_feature_time and calculate_feature_freq, two basic feature sets are calculated. The first, time‐domain feature set, includes mean, variance, standard deviation, max, min, range, and ODBA, where ODBA is short for Overall Dynamic Body Acceleration. This value has been proven to be correlated with the animal's energy expenditure (Wilson et al., 2019). These features are calculated for each ACC axis separately (denoted with prefix x, y, z in the output data frame), except for ODBA, which is calculated using all available axes. The frequency‐domain feature set includes main frequency, main amplitude, and frequency entropy. Also, these features are calculated for each ACC axis separately (denoted with prefix x, y, z). Calculations of these features are based on Fast Fourier Transformation (FFT) of ACC data. Frequency entropy here measures unpredictability of the signal. It is worth noting that some ACC datasets may not have a high enough sampling frequency to log useful frequency information (Nathan et al., 2012). For example, Gilbert et al. (2016) studied white storks using ACC data with a 1 Hz sampling rate, which is insufficient to accurately register the stork's wingbeat frequency, while in our white stork example the sampling frequency of 10.54 Hz could accurately assess wingbeat frequency at 3.1 Hz. Thus, if sampling frequency is low, it is better not to use frequency‐domain features for behavior classification. In addition, it should be considered that the functions calculate_feature_time and calculate_feature_freq provide an essential but not an exhaustive list of potential features. Since it has been asserted that feature engineering can improve the performance of machine learning models (Boehmke & Greenwell, 2019), users may consider calculation of custom features. All functions in the rabc package are also able to process custom features after the user has included these in the feature data frame using functions cbind or bind_cols from the “dplyr” package (Wickham et al., 2021).

In the following, we present the relevant R code calculating features from ACC data:

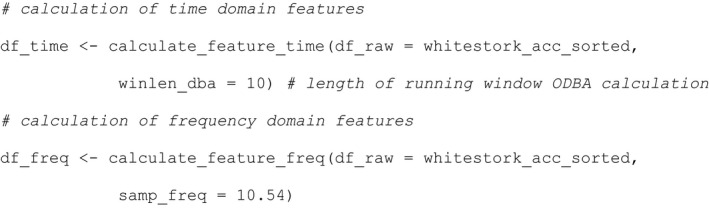

### 2.4 Feature selection

Feature selection is the process of selecting a subset of relevant features for use in model building (Chakravarty et al., 2019). In animal behavior studies using ACC, dozens of features are typically used in model building (e.g., Shamoun‐Baranes et al., 2012). Although a relatively small number compared to often hundreds of features were used in human behavior classification models (Zhu et al., 2017), there may still be redundancy in the feature set. This redundancy may for instance be caused by features that show high correlation with other features and are thus likely to contribute similarly to the behavior classification model. Additionally, irrelevant features may exist that hardly contribute to the classification model. Three aims are being served with feature selection in this package. Firstly, less features will make the model easier to interpret. Indeed, there may for instance be biomechanical connections between features and the ultimate classification model (e.g., Chakravarty et al., 2019). Secondly, fewer features reduce the risk of overfitting and may therewith lead to better behavior classification from ACC data. Thirdly and finally, because of lower computational requirements in assessing behavior from ACC data, reduced feature sets have greater potential to be calculated on‐board the ACC devices themselves, for example, on‐board of light‐weight tracking devices (e.g., Korpela et al., 2020; Nuijten et al., 2020) on which they can either be stored or relayed to receiving stations.

The rabc package's select_features function uses a combination of a filter and a wrapper feature selection method. The filter part removes any redundant features based on the absolute values of the pair‐wise correlation coefficients between features. If two features have a high correlation, the function looks at the absolute correlation of each of the two features with all other features and removes the feature with the largest mean absolute correlation value. The threshold correlation coefficient (cutoff) is user‐defined with a default "cutoff = 0.9". The select_features function will result in a list of features where all feature correlations fall below the threshold correlation coefficient. In the default constellation, the filter function is turned off (i.e., "filter = FALSE").

The purpose of the wrapper is to select most relevant features. The wrapper part applies stepwise forward selection (SFS) (Rückstieß et al., 2011) using the extreme gradient boosting (XGBoost) model, which is not only used for feature selection but also for the final classification model (see below). XGBoost is a scalable tree boosting method that proved to be faster and have a better performance than other currently available tree boosting methods (Chen & Guestrin, 2016). In a comparison with three other supervised machine learning methods (support vector machine, artificial neural network, and random forest models), XGBoost classified behavior from ACC data similarly well to the alternative methods. However, XGBoost had the fastest runtime and the second smallest memory usage (Yu et al., 2021). The default limit to the number of features (no_features) is 5 but can be user defined. The no_features also determines how many rounds of SFS are being conducted. In the first round, each feature is individually used to train a classification model by XGBoost. The feature with highest overall accuracy will be kept into the selected feature set. Then, in the second round, each remaining feature will be combined with the first selected feature to train a classification model and the pair with the highest accuracy will be kept into the selected feature set. This process continues, each round yielding an additional feature on top of the features already selected in previous rounds. This process will stop when the number of rounds equals the no_features setting.

**FIGURE 3:**
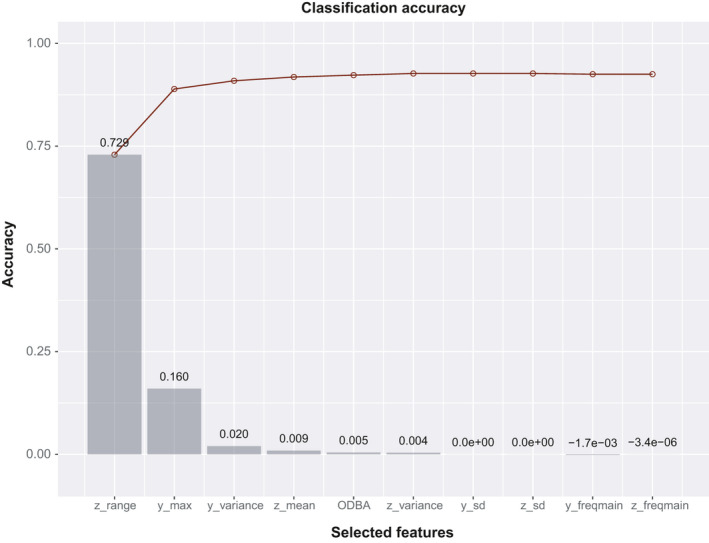
Classification accuracy plot providing an overview of the individual (gray bars) and cumulative (red line and circles) contribution of each feature (in which they were selected in the stepwise forward selection (SFS) process)

The select_features function will return a list, of which the first member (i.e.,.[[1]]) contains a matrix providing the classification accuracy for each of the features (columns) across all steps (rows, top row being the first step) of the SFS process. Once a feature is selected into the selected feature set, the remaining values in this feature's column are set to zero. The second member of the list (i.e.,.[[2]]) contains the names of the selected features in the order in which they were selected in the SFS process. The development of the classification accuracy with each step in the SFS process is plotted with function plot_selection_accuracy (Figure 3). In the case of the white stork dataset, we can see that after the sixth selected feature, “z_variance”, there is almost no further improvement in classification accuracy with the addition of more features.

The relevant R code for feature selection:

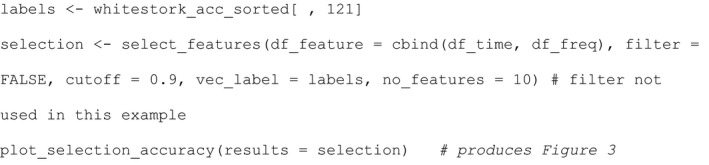

### 2.5 Feature visualization

Above, under “Feature selection” we already mentioned the three objectives with feature selection: improving interpretability, reducing overfitting, and reducing computational requirements. Visualization of the features can further assist in deciding on the features to use in the ultimate behavior classification model, yet its main use is in deciding if any behavior types should be combined to ultimately improve behavior classification performance. Alternatively, the visualization may also lead to considering splitting up existing behavior types into multiple behaviors. In other words, this visualization aids in evaluating the behavior set.

**FIGURE 4:**
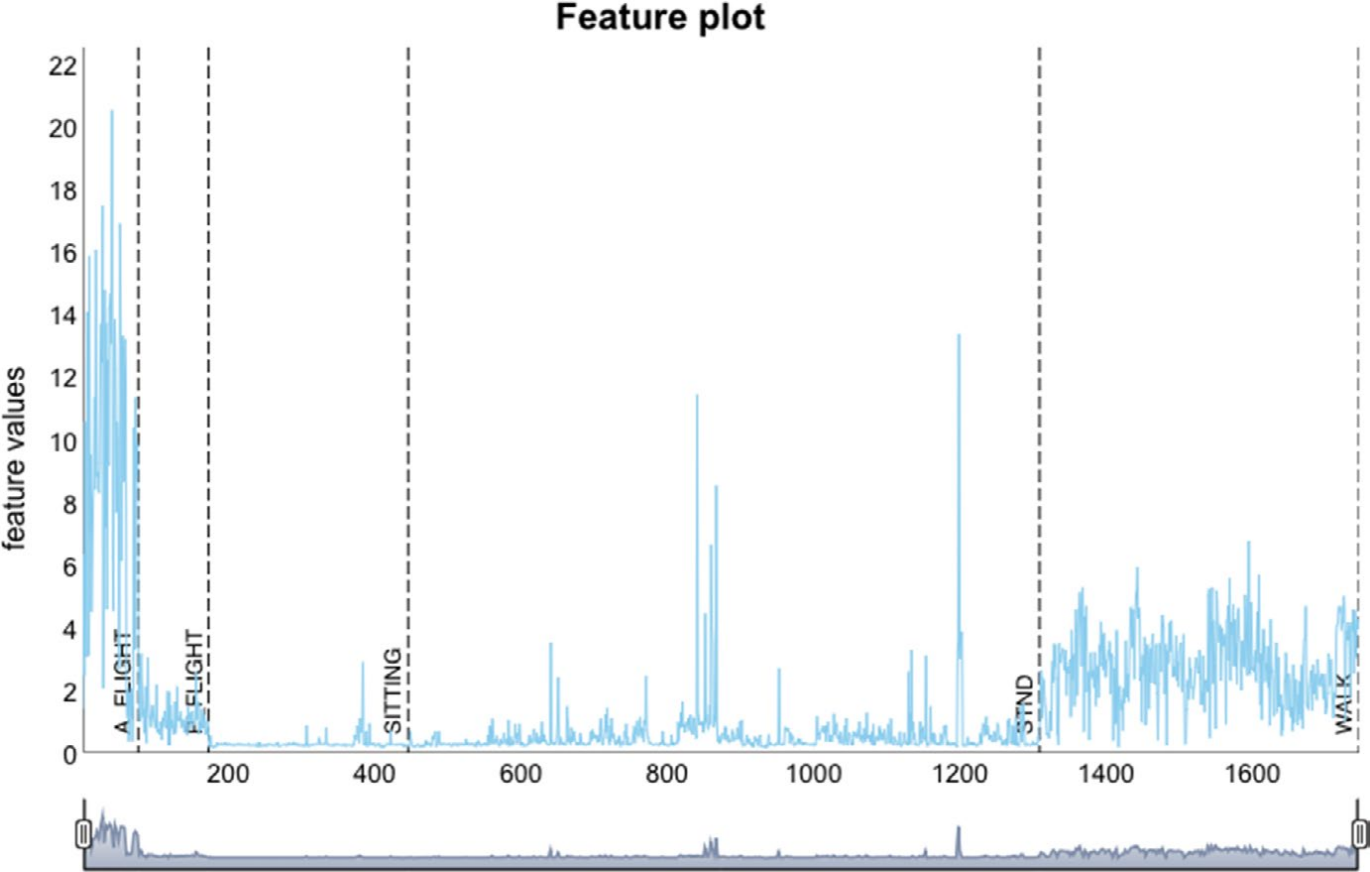
Feature data visualization using a dynamic graph. The feature ODBA in this plot is calculated by function calculate_feature_time. The x‐axis shows the segment numbers of the features ordered by behavior. Vertical dashed lines separate different behavior types

**FIGURE 5:**
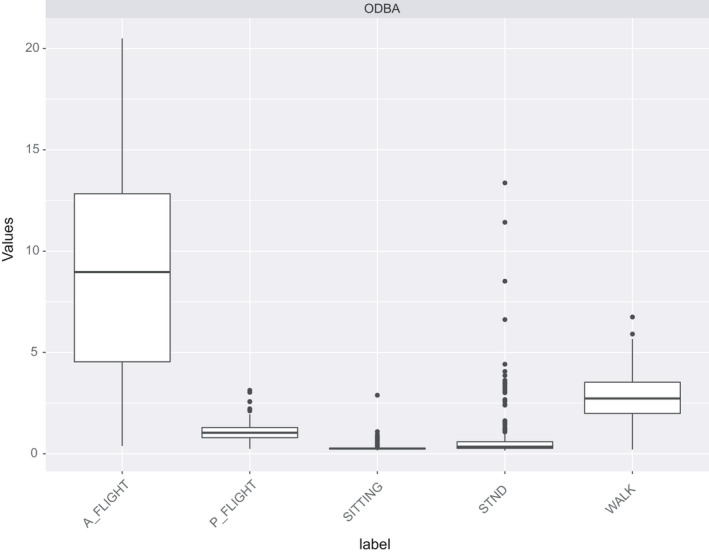
Boxplot of the feature ODBA

The rabc package offers three ways to visualize features. The first two visualize the features in isolation whereas the third is an integrative approach where entire feature domains are analyzed collectively. The first of the visualization methods, plot_feature, draws individual values of features ordered by behavior (Figure 4). The second, plot_grouped_feature, produces a boxplot of a selected feature for all behavior types, as demonstrated for the ODBA feature in Figure 5. In the case of the white stork dataset, it suggests clear differentiation of behaviors by ODBA with a trend of ODBA decreasing from active flight via walking to passive flight, standing, and sitting. The third and most important, integrative approach uses Uniform Manifold Approximation and Projection (UMAP).

In the rabc package, we use UMAP (Konopka, 2020) to plot the different behaviors, represented by differently colored symbols in the two‐dimensional space. UMAP is a very powerful nonlinear dimensionality‐reduction technique, which is also highly suitable for high‐dimensional data visualization (McInnes et al., 2018) and we will here use it to transform and visualize collections of features in a two‐dimensional plot. UMAP has already found its niches in bioinformatics, material sciences, and machine learning (McInnes et al., 2018). Within the broad field of biology, it has been used in bioacoustics studies (e.g., Sainburg et al., 2019), but it has rarely been used in animal behavior studies. The optimal scenario to which one strives is to obtain a representation where each behavior forms an isolated cluster of symbols within this two‐dimensional space. In this way, UMAP provides an indication of how the final classification model will perform, isolated behavior clusters indicating high classification accuracy. If overlaps in clusters exists, researchers may wish to consider grouping certain behaviors because they may not be adequately separated using ACC data. Conversely, if behaviors are spread out over a plot, having those behaviors reclassified in multiple behavior types may be a possibility.

**FIGURE 6:**
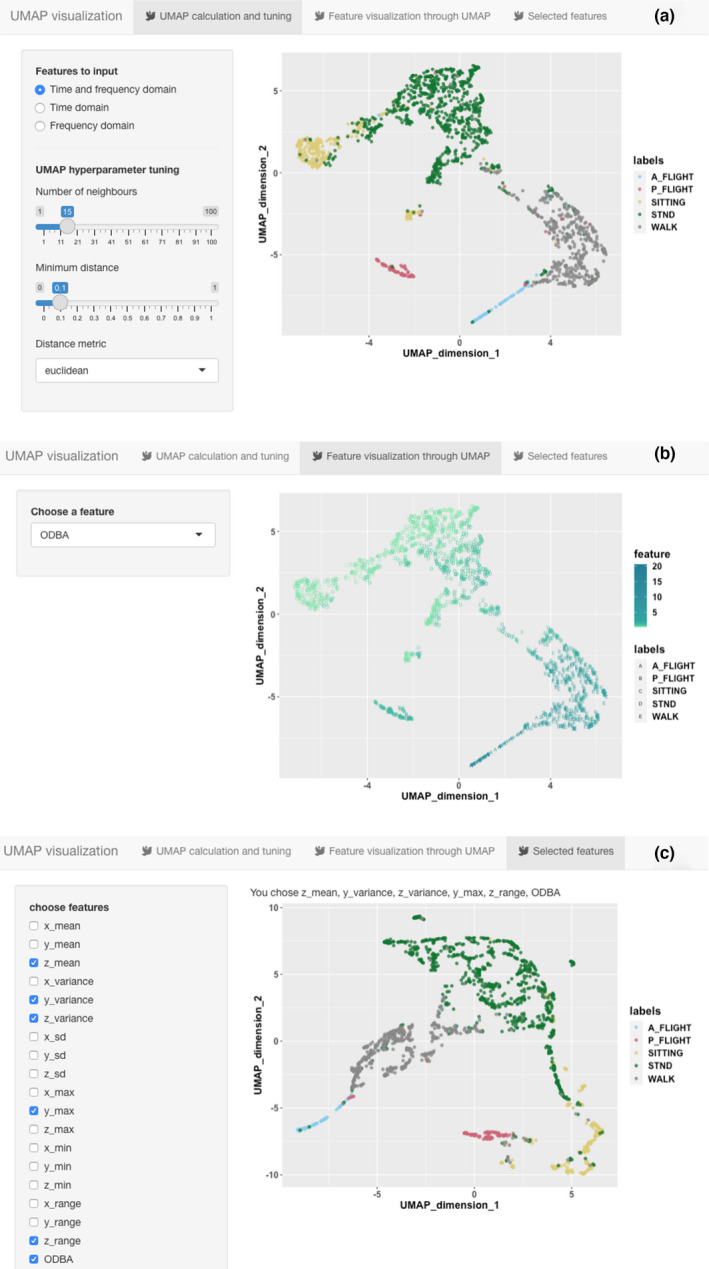
Demonstrations of the three tabs generated by the plot_UMAP function. Tab a—UMAP calculation and tuning—evaluates whether ACC features represent behaviors. The “Features to input” section allows users to choose which feature groups to use as input to UMAP. The “UMAP hyperparameter tuning” section allows users to interactively adjust three hyperparameters within the UMAP function to control the two‐dimensional clustering. Tab b—Feature visualization through UMAP—shows how feature values vary across the two‐dimensional UMAP plot. Users can choose which feature to plot by selecting from the drop box. Tab c—Selected features—allows evaluating the performance of selected features in differentiating between the different behaviors. Users can choose which features to input into UMAP by ticking the checkboxes

We made the UMAP visualization into a Shiny App to facilitate user interaction. The Shiny App was built with the “shiny” R package (Chang et al., 2021). The Shiny App offers an interactive way for users to adjust parameters and update results without the need to rerun code from the R console. There are three tabs in the Shiny App, representing three functions. Tab 1: "UMAP calculation and tuning"—assists with evaluating whether ACC features adequately represent behaviors. Tab 2: "Feature visualization through UMAP"—can show how feature values vary across the two‐dimensional UMAP plot. Tab 3: "Selected features"—assists with evaluating the performance of selected features in differentiating between the different behaviors. In Figure 6, we show screenshots of the three UMAP tabs, loaded with the time and frequency‐domain features from the white stork dataset. It shows that the different behaviors separate generally well (Figure 6a), suggesting that there is good potential to develop a satisfactory performance behavior classification model. In the next tab (Figure 6b), we selected the ODBA feature, the plot showing how its value varies across the different behavior types with active flight having distinguishably high ODBA values followed by walking, then passive flight, standing, and sitting. Finally, in the third tab (Figure 6c), we only selected the six features identified by function select_features to form a new UMAP plot. We can see that these features can preserve the manifold structure of the different behaviors. The demo of this Shiny App can be accessed through <https://huiyu‐deakin.shinyapps.io/rabc_UMAP/>.

Presenting the relevant R code for the plotting of features:

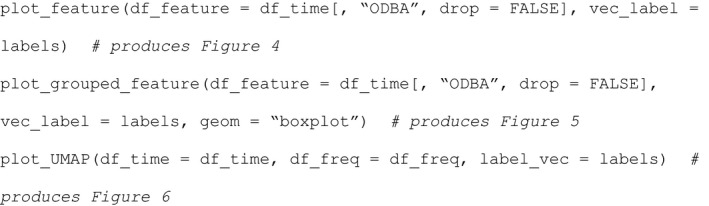

### 2.6 Model training and validation

After feature selection and visualization (including potential grouping and/or splitting of behavior types in the original behavior set), the user can train a supervised machine learning model (XGBoost in this package) with the selected, most relevant features through function train_model. Usually, the construction and evaluation of supervised machine learning models includes three steps: (a) machine learning model hyperparameter tuning by cross‐validation, (b) model training with the optimal hyperparameter set, and (c) evaluating model performance through validation with a test dataset. Function train_model is a wrapper function that utilizes relevant functions from the “caret” (Kuhn, 2020) and “xgboost” packages (Chen et al., 2021) to automatically conduct the three above steps for model construction and evaluation.

Four arguments can be set in the function train_model to control the training and validation processes. Which features to use for model building is set by "*df*", which in the following example is set to “selection$features[1:6]” (i.e., the first six selected features from the feature selection procedure). The “vec_label” argument is used to pass on a vector of behavior types. How to select the hyperparameter set is set by “hyper_choice”, which has two options. The first is "defaults" which will let XGBoost use its default hyperparameters with a fixed setting of “nrounds = 10”. The alternative “hyper_choice” option is "tune", which will run repeated cross‐validations (main parameters: method = “repeatedcv”, number = 5, repeats = 3) to find a best set. Note that for four hyperparameters, a set of alternative values are provided which will be optimized in this procedure (nrounds = c(5, 10, 50, 100), max_depth = c(2, 3, 4, 5, 6), eta = c(0.01, 0.1, 0.2, 0.3), gamma = c(0, 0.1, 0.5)), while for three hyperparameters we fixed the setting (colsample_bytree = 1, min_child_weight = 1, subsample = 1). The settings for these seven hyperparameters are based on our previous experience with a range of different ACC datasets (Yu et al., 2021). For details on the function of the hyperparameters, please refer to https://xgboost.readthedocs.io/en/latest/parameter.html. Finally, “train_ratio” determines the percentage of data used to train the model, the remainder of the data being used for model validation.

The ultimate output consists of four parts. The first is a confusion matrix, depicting how well the ultimate behavior classification model predicts the different behaviors based on the validation part of the dataset only (i.e., 25% of the dataset in our stork example using a train_ratio of 0.75). On the diagonal of this table, where the observed behavior is organized in columns and the predicted behavior is organized in rows, the correct predictions are depicted, with all the wrong predictions being off the diagonal. The overall performance statistics are presented next, the meaning of which is explained in detail in <https://topepo.github.io/caret/measuring‐performance.html>. The third part of the output, statistics by class, presents a range of performance statistics for the individual behavioral categories, which are explained in detail in <https://topepo.github.io/caret/measuring‐performance.html>. Finally, the importance of the various features in producing the behavior classification model is being presented.

**FIGURE 7:**
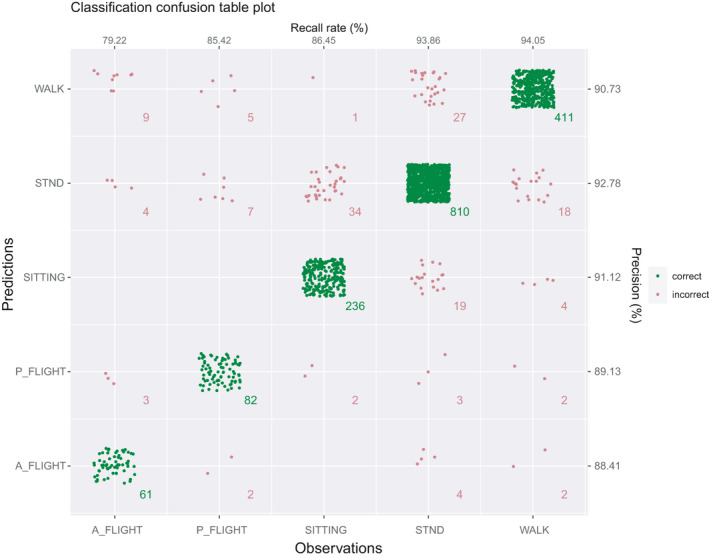
Confusion matrix plot of fivefold cross‐validation results. The dots in the graph are colored according to the classification results, with blue and red symbols being correct and incorrect classifications, respectively. Sample size for each observation and prediction combination is provided

Another way of calculating and visualizing the performance of the behavioral classification model makes use of cross‐validation using function plot_confusion_matrix. In this case, the entire dataset is randomly partitioned into five parts. In five consecutive steps, each of the five parts is used as a validation set, while the remaining four parts are used for model training. This procedure thus resembles a fivefold “classification model training and validation” with a train_ratio of 0.8, be that in this case the dataset is systematically divided and each point in the dataset is being used for the validation process at some point (see function createFolds in “caret” for more details). Thus, after all five training and validation rounds, all behavioral observations will also have an associated predicted behavior, which are being stored in the data frame that is being returned by plot_confusion_matrix in addition to a plot of the confusion table (Figure 7).

The relevant R code for classification model training and validation:

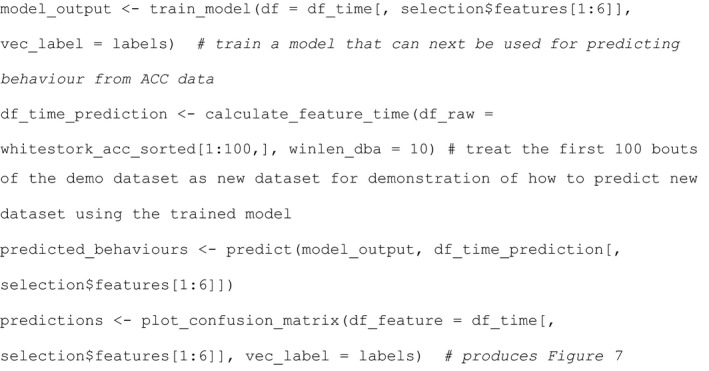

### 2.7 Classification result check

**FIGURE 8:**
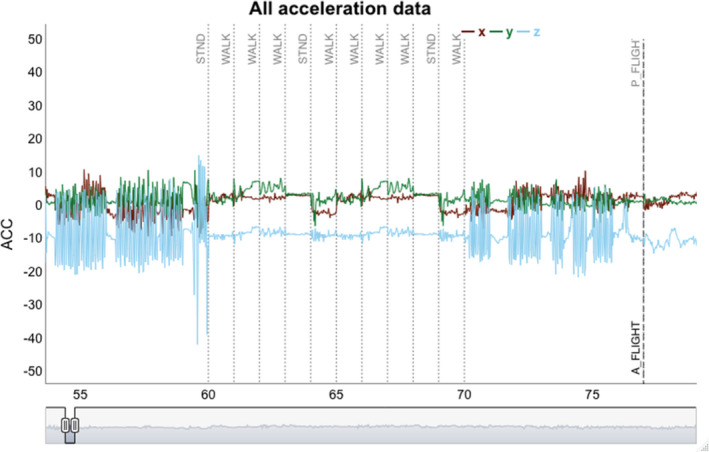
ACC data visualization including behavior classification results using a dynamic graph. White stork ACC data are shown from segment 55 to 80 (cf Figure 2b). Vertical black, dashed lines separate different originally observed behavior types, while vertical gray dotted lines mark incorrect predictions with predicted behavior type labeled at the top

Using the predictions from the behavior classification model, we can now return to the original ACC data to evaluate which ACC signals lead to correct and incorrect classifications using function plot_confusion_matrix. This function basically uses the same digraph with near identical look to function plot_acc used earlier. The only deviation is that all incorrect predictions (identified using the data frame from function plot_confusion_matrix) are now marked as such. The original behaviors are grouped and separated by dashed lines with the corresponding original behavior stated at the base of the dashed lines. The incorrect predictions are marked by dotted lines with the predicted behavior stated at the top (Figure 8).

The R code to visualize incorrect classifications

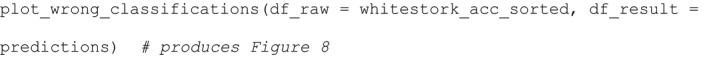

## 3 DISCUSSION AND CONCLUSIONS

As demonstrated, the rabc package can assist researchers in developing good animal behavior classification models in an interactive fashion. ACC data visualization assists in the detection of aberrant associated behavior scores. Feature visualization helps researchers to understand how different features distribute across behaviors and whether the current behavior set potentially needs adjustments, either by grouping or by splitting behaviors into new behavior types. Finally, classification‐result visualization assists the understanding of misclassification patterns. Other than the visualization functionalities, this package provides complete functions to perform behavior classification through XGboost, including feature calculation, feature selection, model hyperparameter tuning, model training and validation, and an output classifier for future ACC data classification.

Given its unique aim and functionality, the rabc package will be a valuable addition to the growing array of R packages already available for behavior and movement analyses (Joo et al., 2020). There is one other R package, “m2b”, that shows some resemblance to the rabc package in that it uses supervised machine learning (random forest) to classify behaviors, be it from GPS rather than ACC data. The rabc package only supports classification in a supervised fashion, which requires users to label ACC data with the corresponding behavior types. However, in some cases behavioral data may not be available and for those circumstances users may want to resort to using the Ethographer package in Igor Pro (WaveMetrics Inc., USA) for processing ACC data in an unsupervised fashion (e.g., Berlincourt et al., 2015).

A non‐R tool designed for animal behavior classification that also uses ACC data in combination with behavioral observations is AcceleRater. Like rabc, AcceleRater trains behavior classification models, yet, there are three major differences between AcceleRater and the rabc package. Firstly, the rabc package is written in R and used in the R environment, which gives users ample freedom of preprocessing and postprocessing the data. Secondly, rather than offering a “black‐box” training process, the visualization tools within rabc assist users in building an understanding of the behavior classification process and why some behaviors can be better classified than others, providing avenues to modify or improve the behavior classification model. Finally, the classification model trained in rabc can be exported and used on‐board of trackers as for instance used in (Yu et al., 2021). It is worth noting that the features calculated in the rabc package can be further extended if deemed necessary. Users can develop additional features and include these in the here described analyses and the ultimate generation of a behavior classification model. Although we only use XGboost as the supervised machine learning model in this package, users can potentially use the output from the rabc package as input to the “caret” package. This will allow for the use of other machine learning models in generating behavior classification models such as decision tree, support vector machine, and random forest. Finally, although ACC data from different animal species and under a variety of circumstances are increasingly becoming available, where possible, we encourage making these accessible with the associated behavior labels. Such data may not only be used to guide studies on new species with comparable behavioral repertoires, but also have the potential to ultimately generate cross‐species behavioral classification models.

## CONFLICT OF INTEREST

The authors declare that there is no conflict of interest.

## AUTHOR CONTRIBUTIONS

**Hui Yu:** Conceptualization (equal); Methodology (lead); Software (lead); Validation (lead); Writing‐original draft (lead). **Marcel Klaassen:** Conceptualization (equal); Methodology (supporting); Software (supporting); Writing‐review & editing (lead).

## Data Availability

The white stork dataset used in this paper is accessible from the online software AcceleRater website: http://accapp.move‐ecol‐minerva.huji.ac.il/. The dataset is also archived at: https://doi.org/10.5061/dryad.dz08kprxv.
